# Oestrogen Activates the MAP3K1 Cascade and β-Catenin to Promote Granulosa-like Cell Fate in a Human Testis-Derived Cell Line

**DOI:** 10.3390/ijms221810046

**Published:** 2021-09-17

**Authors:** Melanie K. Stewart, Pascal Bernard, Ching-Seng Ang, Deidre M. Mattiske, Andrew J. Pask

**Affiliations:** 1 School of BioSciences, The University of Melbourne, Melbourne, VIC 3010, Australia; stemk@student.unimelb.edu.au (M.K.S.); pascal.bernard@unimelb.edu.au (P.B.); deidre.mattiske@unimelb.edu.au (D.M.M.); 2 Melbourne Mass Spectrometry and Proteomics Facility (MMSPF), The Bio21 Molecular Science and Biotechnology Institute, The University of Melbourne, Melbourne, VIC 3010, Australia; ching-seng.ang@unimelb.edu.au

**Keywords:** oestrogen, SOX9, β-catenin, MAP3K1, gonad differentiation

## Abstract

Sex determination triggers the differentiation of the bi-potential gonad into either an ovary or testis. In non-mammalian vertebrates, the presence or absence of oestrogen dictates gonad differentiation, while in mammals, this mechanism has been supplanted by the testis-determining gene *SRY*. Exogenous oestrogen can override this genetic trigger to shift somatic cell fate in the gonad towards ovarian developmental pathways by limiting the bioavailability of the key testis factor SOX9 within somatic cells. Our previous work has implicated the MAPK pathway in mediating the rapid cellular response to oestrogen. We performed proteomic and phosphoproteomic analyses to investigate the precise mechanism through which oestrogen impacts these pathways to activate β-catenin—a factor essential for ovarian development. We show that oestrogen can activate β-catenin within 30 min, concomitant with the cytoplasmic retention of SOX9. This occurs through changes to the MAP3K1 cascade, suggesting this pathway is a mechanism through which oestrogen influences gonad somatic cell fate. We demonstrate that oestrogen can promote the shift from SOX9 pro-testis activity to β-catenin pro-ovary activity through activation of MAP3K1. Our findings define a previously unknown mechanism through which oestrogen can promote a switch in gonad somatic cell fate and provided novel insights into the impacts of exogenous oestrogen exposure on the testis.

## 1. Introduction

Sex determination triggers the differentiation of the bi-potential gonad into either an ovary or testis, a process that is critical for establishing the corresponding secondary sex characteristics. In vertebrates, sex can be determined either by environmental or genetic cues and the switch mechanisms are highly variable [1]. In environmental sex determination (ESD), an external cue such as temperature, social changes or pH causes the activation of aromatase (the enzyme that converts testosterone to oestrogen) and triggers the bi-potential gonad to form an ovary [2]. In contrast, in genetic sex determination (GSD), a gene is the critical switch. Despite having a genetic sex determining mechanism, most species with GSD are oestrogen sensitive and exposure during early developmental stages can trigger ovarian development, even in genetic males. Oestrogen-induced sex reversal has been reported in all vertebrate groups [3,4,5,6,7,8,9], suggesting a fundamental and conserved role for oestrogen in modulating the core pathways required for gonad determination.

In mammals, *SRY* on the Y chromosome directs the differentiation of a testis through the activation of the transcription factor SOX9 [10,11]. SOX9 is both necessary and sufficient to drive testis development [12,13]. At embryonic (E) day 10.5 of mouse development, SOX9 is initially present in the cytoplasm of XX and XY somatic cells in the bipotential gonads [14]. Upon peak *Sry* expression in XY gonads, SOX9 is rapidly translocated to the nucleus where it activates expression of the key testis developmental genes *Amh, Fgf9* and *Ptgds* [15]. In the absence of *Sry,* the cytoplasmic pool of SOX9 disappears [14] and subsequent expression of *Rspo1, Ctnnb1, Wnt4* and *FoxL2* further suppresses the male developmental pathway to trigger formation of an ovary [15]. *Ctnnb1* encodes β-catenin, a Wnt signalling protein that is activated by *Rspo1* in an ovary-specific manner from E12.5 [16]. Interestingly, the ectopic stabilisation of β-catenin in mice can inhibit expression of *Sox9* and *Amh* [17] and cause male-to-female sex reversal [18], demonstrating its ability to suppress the male developmental program and promote ovarian development. There exists an inherent antagonism between these pro-testis and pro-ovary factors, where the stabilisation of β-catenin can promote ovarian development and suppress testis formation while the nuclear translocation of SOX9 is essential for testis development and suppresses ovarian pathways. Indeed, mutations affecting SOX9 nuclear import can cause XY gonadal dysgenesis in humans [19,20], demonstrating this is an integral step for mammalian testis development to proceed.

While oestrogen is not intrinsically required for early mammalian ovarian development—as demonstrated in mice [21,22]—exogenous exposure of the bi-potential gonad to oestrogen can suppress male developmental pathways in marsupials [23]. Oestrogen blocks nuclear translocation of SOX9 in mammalian Sertoli cells, leading to decreased expression of SOX9 target genes that are essential for male development, while promoting activation of ovarian genes [23]. We have demonstrated this mechanism of oestrogen action in the human testis-derived cell line NT2/D1 [24] and in tammar wallaby, where it resulted in male-to-female sex reversal [23]. Together, this suggests that the mechanisms by which oestrogen drives ovarian development in non-mammalian vertebrates have remained intact in mammals.

We previously demonstrated that oestrogen causes cytoplasmic retention of SOX9 via non-genomic signalling cascades that rapidly activate ERK1/2 (a member of the mitogen activated protein kinase (MAPK) family) and promote the stabilisation of microtubules [25]. ERK1/2 is known to be oestrogen responsive across a broad range of cell types [26,27,28,29] and its activation is also observed in MAP3K1 gain-of-function mutations that are associated with XY gonadal dysgenesis in humans [30,31]. Therefore, the MAP3K1 cascade acts as a key mediator of the antagonistic ovary and testis developmental pathways in gonad somatic cells. Increased MAP3K1 activity leads to hyper-phosphorylation of downstream MAPKs ERK1/2 and p38, alongside increased binding to RHOA, ROCK, and AXIN [32]. Gain-of-function mutations in MAP3K1 can be rescued by increased activity of another MAP3K, MAP3K4 [32], which is required for the initial expression of *Sry* in mice [33]. Thus, the activation of the MAP3K1 cascade presents as a possible mechanism through which oestrogen can activate ERK1/2 to mediate a cell fate shift from pro-testis (SOX9) to pro-ovarian pathways (β-catenin/WNT4).

The ability of oestrogen to promote the tilt towards ovarian developmental pathways not only relies on the suppression of SOX9 but also the subsequent activation of ovarian genes. We have confirmed the activation of *FOXL2* and *WNT4* following exogenous oestrogen treatment in wallaby XY gonads and human NT2/D1 cells [23]; however, it has not been established if oestrogen can promote β-catenin stabilisation in the testis. Long-term oestrogen treatment can increase *Ctnnb1* transcription in the mouse prostate [34] and uterus [35] and trigger its nuclear localisation in human endometrial cells [36]. β-catenin activity is also dependent on its phosphorylation status and can therefore be rapidly influenced by non-genomic oestrogen receptor signalling. The most well characterised of these is phosphorylation of Ser33/Ser37/Thr41 by GSK3β [37], which leads to the ubiquitination and degradation of β-catenin [38]. β-catenin can also be phosphorylated at Ser552 by AKT and PKA [39,40], which promotes its transcriptional activity. While there is currently no evidence that oestrogen can directly stimulate phosphorylation of β-catenin, oestrogen can activate its protein kinases, including AKT in a breast cancer cell lines [41] and neurons [42], and PKA in testicular germ cell cancers [43]. Together, this suggests oestrogen could regulate β-catenin activity through the activation of kinases; however, the action of oestrogen on these kinases in the gonads is unknown.

Here, we examine the impact of short- or long-term exposure to oestrogen on the MAP3K1 and MAP3K4 cascades by proteomics and phosphoproteomics to determine if oestrogen can cause a shift from pro-testis to pro-ovary gene regulatory networks through these pathways. From these investigations, we have defined a previously unknown mechanism through which oestrogen can mediate gonad somatic cell determination pathways and provided novel insights into the response of testis cells to exogenous oestrogen.

## 2. Results

### 2.1. EE2 Treatment Causes Non-Genomic and Genomic Changes in NT2/D1 Cells

**Figure 1:**
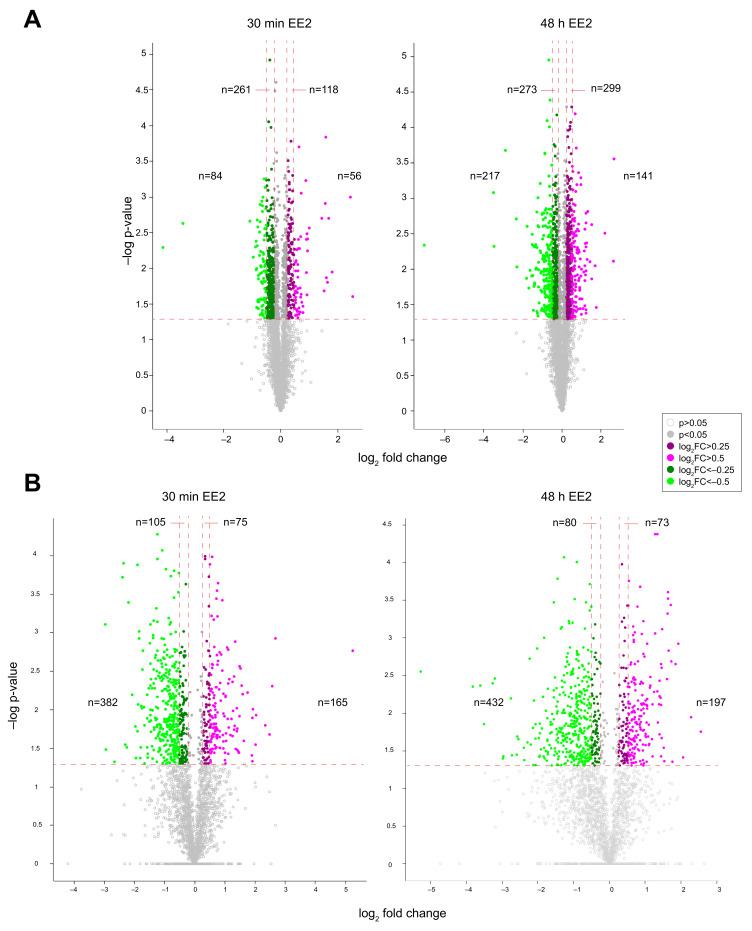
Volcano plots of proteomic (**A**) and phosphoproteomic (**B**) changes following 100 nM EE2 for 30 min or 48 h. *p* = 0.05 is indicated by the horizontal dotted red line, open circle plot points are non-significant (*p* > 0.05) and closed circle plot points are significant (*p* < 0.05). The log_2_ fold change (FC) is indicated by the colour of plot points, vertical dotted red lines indicate 0.25 and 0.5 log_2_FC. Purple = log_2_FC > 0.25, pink = log_2_FC > 0.5, dark green = log_2_FC < −0.25, green = log_2_FC < −0.5.

We utilised differential SILAC based mass spectrometry to quantify changes to the proteome and phosphoproteome of NT2/D1 cells after a brief (30 min) or prolonged (48 h) exposure to oestrogen (EE2). We examined significantly changed proteins (*p* < 0.05) with a cut-off of log_2_ fold change (log_2_FC) of 0.25 and 0.5, as this aligns with the subtle changes we have observed in our previous studies in these cells. After brief exposure, we observed 14.2% of quantified proteins changed significantly by a log_2_ fold change (log_2_FC) of 0.25 (either direction; 519 of 3651), while 3.8% changed by a log_2_FC of 0.5 (140 of 3651; Figure 1A), demonstrating that oestrogen can rapidly impact a broad range of pathways after only 30 min. As expected, we observed greater changes after prolonged exposure, where 23.7% of quantified proteins changed significantly by a log_2_FC of 0.25 (930 of 3922) and 9.1% changed by a log_2_FC of 0.5 (358 of 3922; Figure 1A). From our phosphoproteomic data, we identified a total of 1282 phosphorylated proteins, which contained 2595 phosphosites in cells treated with EE2 for 30 min, and 1268 phosphorylated proteins, which contained 2652 phosphosites in cells treated for 48 h (localisation probability ≥ 0.75). Following oestrogen exposure for 30 min, 28% (727 of 2595) of phosphosites significantly changed by a log_2_FC of 0.25, and 21.1% of phosphosites changed by a log_2_FC of 0.5 (547 of 2595). Together, these findings demonstrate that oestrogen can rapidly alter signalling cascades by changing protein phosphorylation. We observed a similar number of phosphosite changes in cells treated for 48 h, where we observed 29.5% (782 of 2652) changed by log_2_FC 0.25 and 23.7% changed by log_2_FC of 0.5 (629 of 2652; Figure 1B).

**Figure 2:**
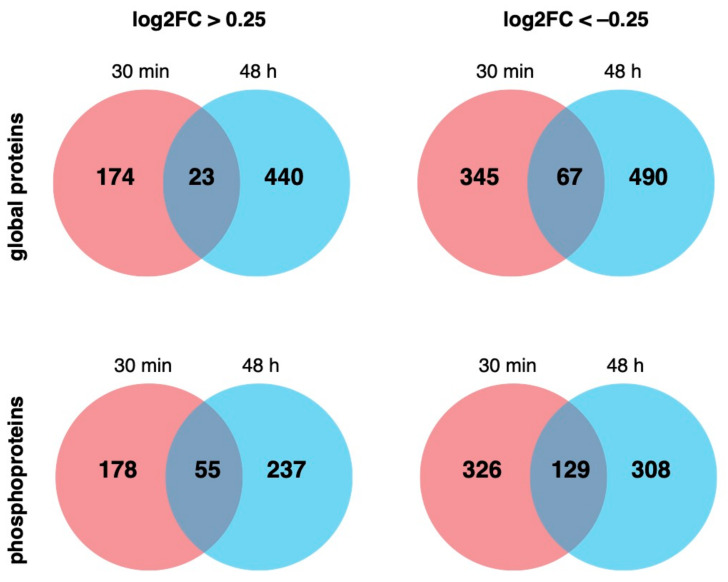
The number of proteins and phosphoproteins significantly upregulated or downregulated after 30 min (red) and 48 h (blue) and those similarly changed in both datasets are shown in the overlapping space.

To examine any consistent changes between timepoints, we determined which proteins had significantly changed both after 30 min and 48 h of EE2 treatment. 23 proteins significantly increased by a log_2_FC of >0.25 and 67 significantly decreased by a log_2_FC of <−0.25 across both datasets (Figure 2). Examination of proteins that had any significant changes to a phosphosite in both datasets revealed 55 proteins with phosphosites that changed a log_2_FC of >0.25 and 129 proteins with phosphosites that changed a log_2_FC of <−0.25 (Figure 2). This demonstrates that some of the non-genomic changes occurring after 30 min of oestrogen were maintained through to 48 h.

**Figure 3:**
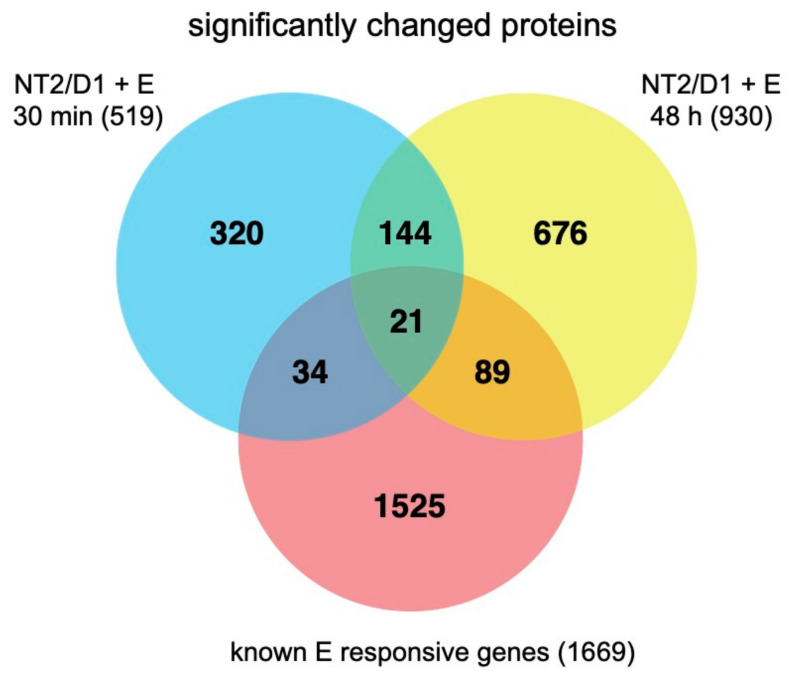
The number of proteins significantly changed in both 30 min (blue) or 48 h (yellow) of EE2 treatment datasets in NT2/D1 cells and their overlap with known oestrogen (E; red) responsive genes.

We next compared our dataset of significantly altered proteins (*p* < 0.05, log_2_FC < −0.25 or >0.25) to the Dragon database of oestrogen responsive genes (ERGs; Figure 3) [44]. Proteins altered after 30 min of oestrogen exposure represent non-genomic targets (as there is not sufficient time for activated ERs to elicit genomic responses), while proteins altered after the 48-h timepoint reflect both non-genomic targets, as well as genomic changes. Thus, the comparison between the ERG list and our two treatment timepoints provides novel insights into the non-genomic and genomic targets of oestrogen signalling. We identified 34 ERGs that overlapped solely with proteins impacted after 30-min of oestrogen exposure (non-genomic targets) and 89 ERGs that overlapped solely with proteins impacted after 48-h of oestrogen exposure (likely genomic targets), while 21 genes overlapped with proteins from both proteomic datasets (additional likely non-genomic targets). We observed 144 proteins overlapping between our two treatment periods that were unique from the ERG dataset, as well as 320 and 676 unique to the 30-min and 48-h treatments, respectively. These provide an expanded list of oestrogen responsive proteins in the human genome. There was still a large proportion of proteins that were unique to each dataset, which is unsurprising given the differences between timepoints in our datasets. Overall, however, it is important to consider that the regulation of proteins versus genes is a major difference when comparing to an ERG list, particularly given that alterations to translation and post-translational modifications can create a myriad of changes between the transcription of a gene and the activity of a protein.

### 2.2. GO Terms Related to the Cytoskeleton and RNA Processing Are Enriched Following EE2 Treatment

**Figure 4:**
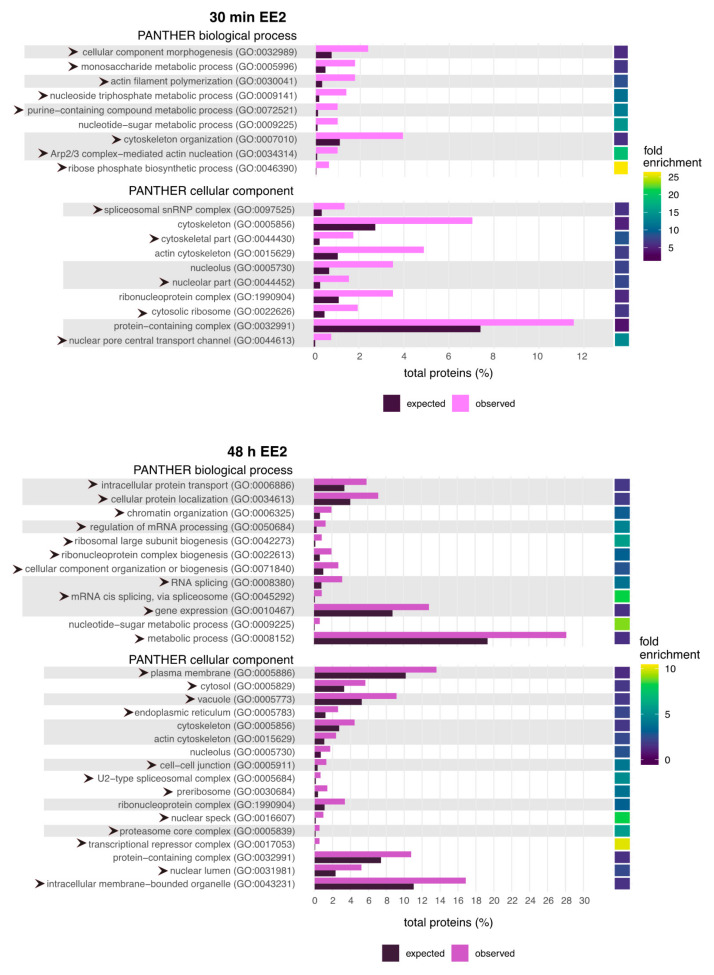
PANTHER Gene Ontology (GO) terms significantly enriched in the proteome following 30 min or 48 h of EE2 treatment. Terms clustered together by the grey/white background indicate those related by hierarchy. The horizontal bar plot denotes the percentage total number of expected and observed proteins for PANTHER biological processes and cellular components that were significantly enriched (Fishers exact test; corresponding fold enrichment show on the far right) following either 30 min (**top**) or 48 h (**bottom**) of EE2 treatment.

**Figure 5:**
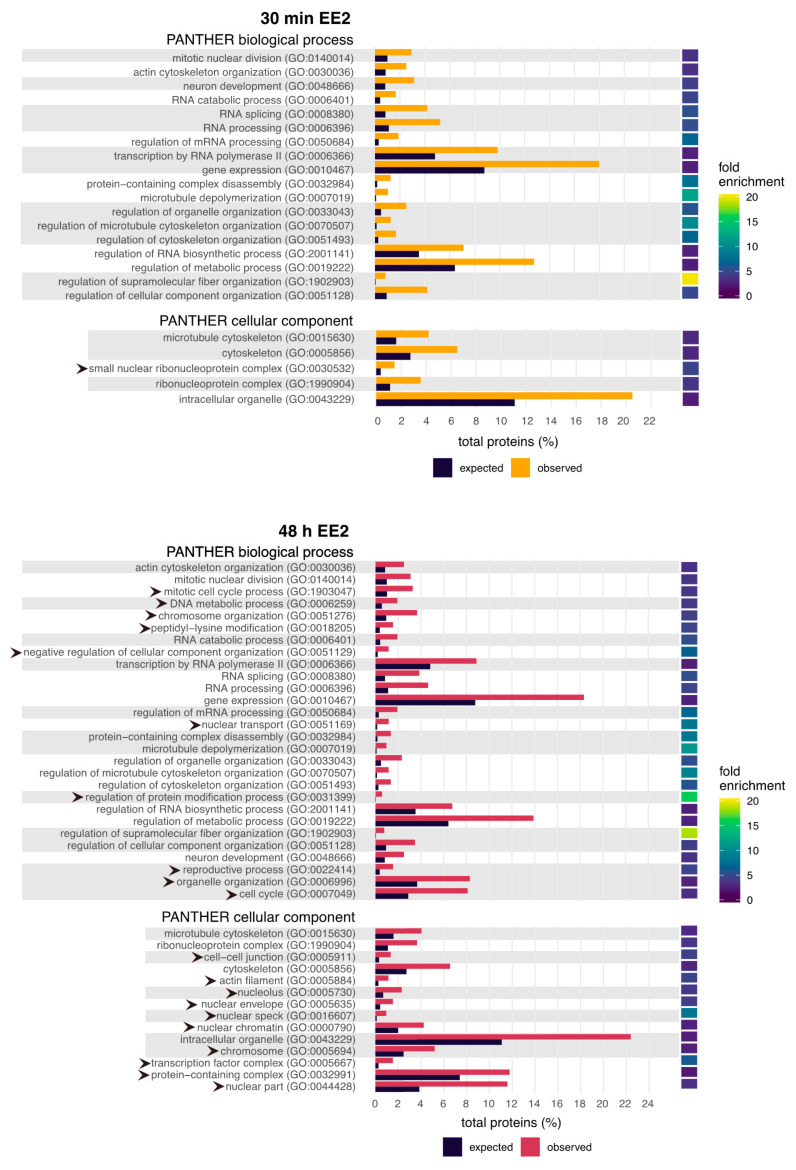
PANTHER Gene Ontology (GO) terms significantly enriched in the phosphoproteome following 30 min or 48 h of EE2 treatment. Terms clustered together by the grey/white background indicate those related by hierarchy. The horizontal bar plot denotes the percentage total number of expected and observed proteins for PANTHER biological processes and cellular components that were significantly enriched (Fishers exact test; corresponding fold enrichment show on the far right) following either 30 min (**top**) or 48 h (**bottom**) of EE2 treatment.

To understand the global effects of oestrogen treatment on NT2/D1 cells, we performed Gene Ontology (GO) enrichment analysis using PANTHER (http://www.pantherdb.org, accessed on 5 April 2020) [45] on proteins that were significantly changed (*p* < 0.05, log_2_FC > 0.25 or <−0.25) and assessed changes to biological processes and cellular components. The percentage of expected and observed proteins and the enrichment for each corresponding GO term is presented for the global proteome (Figure 4) and phosphoproteome (Figure 5). Specific values can be found in Tables S1 and S2. Analysis of the global proteome after 30 min of EE2 treatment revealed changes primarily to terms related to the cytoskeleton, metabolism, ribosomes/RNA processing and the nuclear pore complex (Figure 4). After 48 h, we observed some changes to the biological processes and cellular components enriched (Figure 4; arrowheads indicate unique terms), including a greater emphasis on terms related to ribosomes, ribonucleoprotein complex and RNA processing, as well as the addition of terms related to intracellular protein transport. GO terms related to the cytoskeleton and metabolic processes were present at both timepoints. Interestingly, pathway enrichment analysis of the phosphoproteome revealed similar GO terms were enriched, including terms related to the cytoskeleton, RNA processing and ribonucleoprotein complex (Figure 5). We observed conservation of these terms from 30 min to 48 h of EE2 treatment and the GO pathway with the greatest enrichment in both phosphoproteome datasets was ‘regulation of supramolecular fibre organisation’ (GO:1902903), a term associated with cytoskeleton regulation. Some unique terms (Figure 5; indicated by arrowheads) were present in the 48-h EE2 treatment, such as ‘reproductive process’ (GO:0022414)—which included the sex differentiation gene ATRX, the gap junction protein GJA1 and the sperm protein NASP—‘cell–cell junction (GO:0005911)’, and others related to transcriptional changes.

### 2.3. EE2 Treatment Leads to Activation of the MAP3K1 Cascade, However, Not the MAP3K4 Cascade

**Figure 6:**
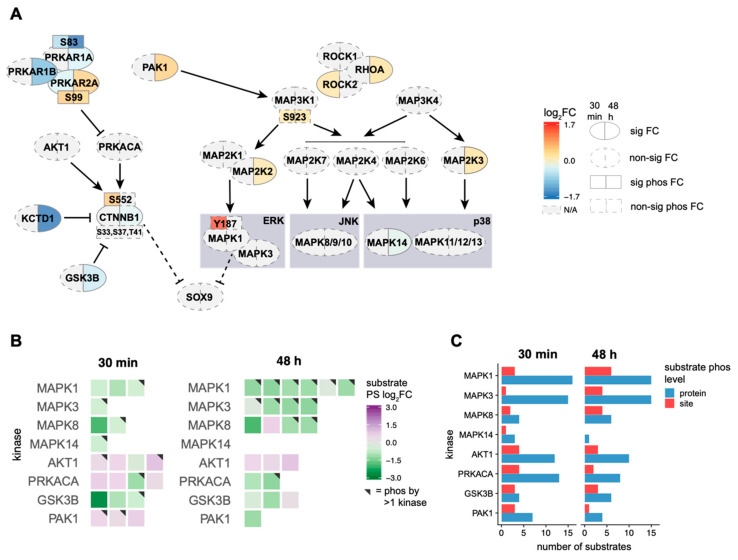
Proteins involved in MAP3K1/MAP3K4 cascades in response to oestrogen and analysis of the activity of key kinases. (**A**) Fold changes to MAP3K4 and MAP3K1 pathways and related proteins following EE2 treatment for 30 min (left half of oval) and 48 h (right half of oval). Significant (*p* < 0.05) fold changes (FC) for proteins (oval) and phosphoproteins (rectangle) are surrounded with a solid line, while non-significant (*p* > 0.05) changes are surrounded with a dashed line. Indirect relationships are indicated by a dashed line. Proteins with no detected significant change are indicated by a grey fill (N/A). EE2 treatment led to upregulation of proteins involved in the MAP3K1 cascade (**B**) Target substrate phosphosite (PS) log_2_FCs for key kinases involved in the MAP3K1/MAP3K4 cascades after 30 min and 48 h of EE2 treatment. Grey triangles in the top right corners of the tiles indicate this substrate phosphosite is phosphorylated by more than one kinase. A decrease in phosphorylation indicates reduced activity of the corresponding kinase. (**C**) Number of substrates with a significant change on either the phosphosite (red) or protein (blue) level for each of the key kinases in the MAP3K1/MAP3K4 cascades following EE2 treatment for 30 min or 48 h. Protein level phosphorylation indicates that the target protein had a change in phosphorylation, but not at the precise phosphosite recorded as being a target of that kinase. A large differential between the number of protein and site level changes may indicate a larger change in kinase activity than was shown in (**B**).

To understand the impact of oestrogen specifically on the MAP3K1 and MAP3K4 cascades and β-catenin—which have a critical role in gonad determination—we examined the members of these cascades and any changes to their abundance or phosphorylation (Figure 6A). Specific log_2_FCs and *p*-values of proteins and phosphosites shown in Figure 6 are presented in Tables S3–S5. Non-significant findings are also included in these analyses where there is relevant biological context.

PAK1 lies upstream of MAP3K1 and was significantly upregulated after 48 h of EE2 treatment. While we detected no change in the abundance of MAP3K1, we observed a non-significant (30 min *p* = 0.129; 48 h *p* = 0.112) increase in phosphorylation of Ser923 at both timepoints. This site is uncharacterised but has been detected in a large number of datasets (www.phosphosite.org, accessed on 5 April 2020), suggesting it may be important in the regulation of MAP3K1 activity. The downstream targets of MAP3K1—MAP2K1 and MAP2K2—increased in abundance after 48 h; however, only MAP2K2 was significant. We also observed an increase in the phosphorylation of MAPK1 (ERK2) at Tyr187 (a site that leads to activation) after 30 min, consistent with our previous findings showing that EE2 activated ERK1/2 in NT2/D1 cells [25]. This was statistically non-significant (*p* = 0.106), although all replicates showed a consistent trend with increasing abundance (raw values for each replicate were log_2_FC 0.95, 0.80, 0.55 and 3.11). The binding partners of MAP3K1, RHOA and its downstream effectors ROCK1 and ROCK2 increased in abundance, suggesting they may be promoting the activation of MAP3K1. RHOA was significantly upregulated after 48 h of EE2 treatment, while ROCK2 significantly increased in abundance after 30 min and upregulated after 48 h (non-significant). ROCK1 increased in abundance after 48 h, however this was not significant. MAP3K4 had no change in abundance or phosphorylation and there was no consistent effect on its downstream targets: MAP2K3 increased in abundance after 48 h and MAP2K6 (also a target of MAP3K1) decreased after 30 min (non-significant), while there was only one change to a downstream p38 MAPK, MAPK14, which was downregulated after 48 h. We detected no significant changes in the phosphorylation of these targets.

We specifically examined β-catenin as we have shown exogenous oestrogen leads to an increase in expression of its downstream targets. β-catenin decreased slightly in abundance after 48 h of EE2 treatment despite a downregulation in two proteins that promote its degradation: GSK3β [38] and KCTD1 [46]. KCTD1 decreased significantly in abundance after 48 h of EE2 treatment, while GSK3β decreased in abundance at both timepoints, but this was only significant after 48 h of EE2 treatment. We did observe a change to the phosphorylation of β-catenin at a known activation site, Ser552. After 30 min of EE2 treatment, the phosphorylation of β-catenin at Ser552 significantly increased in abundance but this was not maintained at 48 h. The upstream kinases of Ser552 are PRKACA (the catalytic subunit of PKA) and AKT1. AKT1 had no change in abundance or phosphorylation, while there was no detectable change in the abundance or phosphorylation of PRKACA, we did detect changes to the regulatory subunits of PKA: PRKAR1A, PRKAR1B and PRKAR2A that inhibit the activity of PKA. Both PRKAR1A, and PRKAR1B were downregulated after 48 h of EE2 treatment and there was reduced phosphorylation of PRKAR1A at Ser83 (an activation site) at both timepoints, indicating it likely had reduced activity. In contrast, PRKAR2A initially decreased in abundance after 30 min of EE2 treatment, but this was reversed after 48 h; furthermore, we detected an increase in its phosphorylation at Ser99 at both timepoints, suggesting its activation.

### 2.4. Oestrogen Alters the Activity of Kinases Involved in the MAP3K1/3K4 Cascades

To further understand the activity of the kinases mentioned above, we assessed the fold changes to phosphorylation of their known target substrates (Figure 6B) and the number of site or protein level changes (Figure 6C). All results presented for Figure 6B,C are significant changes (log_2_FC difference of 0.25, *p* < 0.05) and shown in Tables S4 and S5. Only 5% of the phosphoproteome has identified kinases [47]. Due to this limitation, protein level changes provide an insight into the potential activity of a kinase and are important for further investigation. Although MAPK1 had an increase in phosphorylation at an activation site, its known target substrates showed an overall decrease in phosphorylation at both timepoints. This trend in decreased phosphorylation of target substrates was the same for MAPK3, which together with MAPK1 forms the ERK1/2 complex. Many of the substrates targeted by ERK1/2 were also substrates for other kinases, suggesting the overall decrease may not be due to an alteration in ERK1/2 kinase activity. Despite this, examination of protein level phosphorylation demonstrated that MAPK1 and MAPK3 had the largest number of substrates exhibiting a significant change, suggesting that there is very likely a change to their activity.

MAPK8 (JNK1), which is downstream of both MAP3K1 and MAP3K4, showed no detectable changes; however, its substrates showed a general trend towards decreased phosphorylation. MAPK14 had the smallest number of changes to substrates, consistent with its downregulation after 48 h of EE2 treatment. While AKT1 had no detectable changes to its phosphorylation or abundance, it demonstrated an overall trend towards activation at both timepoints where all but one of its substrates had increased phosphorylation; furthermore, it had a large number of protein-level substrates with altered phosphorylation, suggesting a change to its activity. The catalytic subunit of PKA, PRKACA, triggered increased phosphorylation of three of four target substrates after 30 min. Similar to ERK1/2 and AKT1, there were a large number of protein-level changes to its target substrate phosphorylation, particularly at 30 min, suggesting a potential change in its activity at this timepoint. In combination with the above data on the regulatory subunits of PKA, these results suggest PKA is activated after 30 min of EE2 treatment. GSK3β—the kinase that targets β-catenin for degradation—had three substrates with reduced phosphorylation after 30 min and two with reduced phosphorylation after 48 h, which is consistent with its downregulation following EE2 treatment. Finally, three target substrates of PAK1 demonstrated increased phosphorylation after 30 min, although two of these are not unique to PAK1. Despite being upregulated after 48 h of EE2 treatment, only one substrate of PAK1 had altered phosphorylation at this timepoint, suggesting there was no increase in its kinase activity.

### 2.5. β-Catenin pSer552 Is Localized to the Nucleus Following EE2 Treatment, but Non-Phosphorylated β-Catenin Remains Unchanged

**Figure 7:**
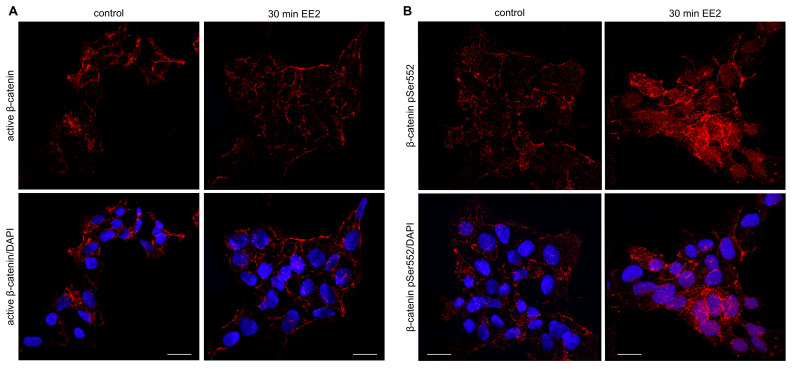
Immunofluorescence staining of active (non-phosphorylated Ser33, Ser37, Thr41) and phosphorylated (pSer552) β-catenin in NT2/D1 cells after 30 min of culture. (**A**) Active β-catenin (red) was present at the cell membrane of both control and 30-min EE2 treated NT2/D1 cells, indicating oestrogen had no effect on its localisation. (**B**) β-catenin pSer552 (red) was present primarily at the cell membrane of control NT2/D1 cells, with a small amount throughout the cytoplasm. NT2/D1 cells treated with 100 nM EE2 for 30 min showed a marked increase in the nuclear (DAPI; blue) localisation of β-catenin pSer552, as well as an increase in the overall signal, demonstrating activation of β-catenin through phosphorylation at this site. Scale bar = 20 μm.

To further characterize the activation of β-catenin detected in our phosphoproteome analyses, we performed immunofluorescence of non-phosphorylated (at Ser33, Ser37 and Thr41) β catenin and β-catenin pSer552. β-catenin phosphorylation at Ser33, Ser37 and Thr41 by GSK3β targets it for degradation and the absence of phosphorylation at these sites is considered ‘active’ β catenin [37]; however, β-catenin can be transcriptionally active following phosphorylation at Ser552 independent of the phosphorylation status at Ser33, Ser37 and Thr41. Examination of non-phosphorylated β-catenin in control and 30-min EE2 treated cells revealed no differences (Figure 7A; visualised in red). In both control and EE2 treated cells, non-phosphorylated β-catenin was present largely at the cell membrane and in the cytoplasm, but not in the nucleus where it activates transcription. These immunofluorescence results confirm what we observed in our mass spectrometry experiments where there was no change to these specific phosphosites following EE2 treatment. We confirmed the activation of β-catenin at Ser552 following oestrogen treatment by immunofluorescence. In control cells, β-catenin pSer552 was present primarily at the cell membrane with a small amount in the cytoplasm (Figure 7B; visualized in red), very similar to the staining of active β-catenin. In contrast, in cells treated with EE2 for 30 min, β-catenin pSer552 increased considerably throughout the cell, and was primarily located in the nucleus, indicating β-catenin may be transcriptionally active following increased phosphorylation at Ser552.

## 3. Discussion

It is well established that oestrogen can influence gonad determination to promote formation of an ovary in a wide range of vertebrates, including mammals [3,4,6,8]. We have previously demonstrated that exposure to oestrogen can cause the cytoplasmic retention of SOX9 in testis cells, leading to a decrease in expression of genes that drive male development and increased expression of pro-ovarian genes [23,24]. We showed that oestrogen treatment rapidly activated ERK1/2 to promote microtubule stabilisation, resulting in the cytoplasmic retention of SOX9 [25]. The MAP3K1/3K4 cascades can activate ERK1/2 and cause a shift from pro-testis (SOX9) to pro-ovarian (β-catenin) activity in the gonads. Thus, we examined the potential for the MAPK pathways to be impacted by rapid and long-term exposure to oestrogen through proteomic and phosphoproteomic analyses.

Gene Ontology (GO) term analyses of our proteomic datasets have demonstrated that oestrogen can target a broad range of processes in testis-derived NT2/D1 cells. We primarily observed changes to pathways related to RNA processing and the cytoskeleton. Oestrogen has previously been implicated in altering pathways related to translation and RNA processing in breast cancer cells [48], particularly PI3K/AKT/mTOR signalling [49]. Interestingly, the mTOR pathway is essential for spermatogenesis and maintaining the blood-testis barrier [50], suggesting oestrogen influence of this pathway could be a mechanism behind its detrimental effect on male fertility. Another key process affected by EE2 treatment was regulation of the cytoskeleton. Our previous research has demonstrated that oestrogen can rapidly target microtubules in NT2/D1 cells to promote cytoplasmic retention of SOX9 [25]—thus, the association of oestrogen treatment with cytoskeleton-related GO terms further reinforces this finding and suggests a broader role for the cytoskeleton in influencing cell fate following oestrogen treatment.

We previously showed oestrogen treatment increases ERK1/2 activity in the testis-derived NT2/D1 cell line and is associated with a shift in the expression profile towards an ovarian cell fate [25]. We confirmed activation of ERK1/2 in our phosphoproteomic data—specifically, increased MAPK1 phosphorylation at Tyr187 after 30 min of EE2 treatment. However, kinase-substrate analyses showed an overall decrease in the phosphorylation of ERK1/2 target substrates. We hypothesise this is because many of the ERK1/2 substrates are known targets of other kinases. Seven of the nine ERK1/2 target substrates are localised to the cytoplasm, suggesting that the decrease in ERK1/2 substrate phosphorylation could be caused by the nuclear accumulation of pERK1/2, resulting in less active ERK1/2 in the cytoplasm. Furthermore, we also observed a large number of protein-level phosphorylation changes of ERK1/2 substrates, and some of these demonstrated increased phosphorylation (data not shown). This suggests that ERK1/2 may be activating other substrates that have been missed in our site-level analyses.

We also observed upregulation of MAP2K2 (MEK2), which lies in between MAP3K1 and ERK1/2, further supporting activation of this cascade. The activation of ERK1/2 mirrors the effects of increased MAP3K1 activity in gain-of-function mutations that are associated with XY gonadal dysgenesis [30,31]; furthermore, we have demonstrated that ERK1/2 mediates oestrogen-induced suppression of SOX9 [25], demonstrating an elegant link between the MAP3K1 cascade and the ability of oestrogen to influence gonad determination pathways. Together, our data suggests activation of the MAP3K1 cascade but no change to the MAP3K4 cascade following oestrogen treatment. Interestingly, this mirrors the results observed in tammar wallaby, where oestrogen treatment of XY gonads led to an increase in transcription of *MAP3K1* but no change to *MAP3K4* [51].

Gain-of-function MAP3K1 mutations that are known to lead to XY gonadal dysgenesis involve the upregulation of RHOA and ROCK1 [30,31,32]—thus, the upregulation of RHOA and ROCKs in our datasets further suggest oestrogen has promoted the activation of MAP3K1. Activation of RHOA in chondrocytes is also associated with cytoplasmic retention of SOX9 and decreased *SOX9* expression [52,53], supporting the link between RHOA upregulation and the suppression of SOX9 by oestrogen in the gonads.

β-catenin is the critical factor that can promote ovarian development downstream of MAP3K1 activation [32] and ectopic stabilisation of β-catenin in mice can induce XY sex reversal through suppression of *Sox9* transcription [18]. Therefore, we examined changes to β-catenin and its regulators to determine if the activation of MAP3K1 had successfully resulted in the activation of this ovarian factor. We observed a reduction in two proteins that promote β-catenin degradation—KCTD1 and GSK3β—following EE2 treatment, suggesting exogenous oestrogen is promoting β-catenin activity by blocking its normal degradation. KCTD1 binds to β-catenin and promotes its cytoplasmic localisation and degradation [46] and GSK3β triggers the destabilisation of β-catenin through phosphorylation at Ser33, Ser37 and Thr41 [38]. Although we observed a downregulation in GSK3β at both timepoints, as well as reduced GSK3β kinase activity, there were no changes to GSK3β-mediated phosphorylation of β-catenin in our mass spectrometry data. Furthermore, we observed no change in the localisation of non-GSK3β-phosphorylated (active) β-catenin in cells treated with EE2 for 30 min. Together, these data suggest exogenous oestrogen did not reduce GSK3β-mediated degradation of β-catenin. Previous research in neurons has demonstrated a transient effect of oestrogen on reducing GSK3β activity [54], suggesting the effect of oestrogen on GSK3β-induced phosphorylation of β-catenin may similarly be a brief occurrence in NT2/D1 cells.

While there was no apparent change to the overall degradation of β-catenin by GSK3β, we did observe activation of β-catenin through significantly increased phosphorylation of Ser552 after 30 min of EE2 treatment and further confirmed this and its nuclear localisation by immunofluorescence. Activation of β-catenin can occur through phosphorylation of Ser552 independently of GSK3β and can promote β-catenin transcriptional activity [39,40]. Indeed, we have previously confirmed that genes downstream of β-catenin activation, *WNT4* and *FOXL2*, show increased expression in NT2/D1 cells following prolonged EE2 exposure [24]. β-catenin can also decrease expression of both *SOX9* and *AMH* in NT2/D1 cells and embryonic mouse gonads [17], an effect we have also reported following exogenous oestrogen treatment [24]. These data suggest that oestrogen treatment does not impact GSK3β-mediated degradation of β-catenin (at these timepoints), but that oestrogen can induce β-catenin to become transcriptionally active independently of this, through phosphorylation at Ser552.

Overall, the activation of β-catenin results in the antagonism of SOX9 and directly promotes expression of ovarian development pathways. It is well known that oestrogen is essential for maintaining granulosa cell fate in adulthood by reinforcing the activation of pro-ovarian factors and suppression of pro-testicular factors [55]. Our findings are in line with the activation of the MAP3K1 cascade and a shift towards a granulosa-like cell fate. It further suggests that the activation of β-catenin by phosphorylation is an important initial response to oestrogen. This oestrogen response is later consolidated by the combined upregulation of both β-catenin and the genomic responses to activated ERα. The activation of β-catenin in Sertoli cells following exogenous oestrogen treatment is a novel finding and refines our understanding of how oestrogen mediates gonad somatic cell fate.

It is unclear which kinase—AKT1 or PKA—is responsible for the phosphorylation of β-catenin at Ser552. Our kinase-substrate analyses suggested that AKT1 and PRKACA (PKA) had increased activity after 30 min of EE2 treatment, and only AKT1 appeared active after 48 h. Interestingly, brief oestrogen treatment of neurons can activate AKT to cause dissociation of β-catenin from its inhibitor GSK3β, eventually leading to decreased activity of GSK3β [56]; this illustrates that there exists a dual action of AKT to promote β-catenin activity by inhibiting GSK3β, as well as phosphorylating β-catenin, and further suggests oestrogen is likely activating AKT in NT2/D1 cells.

The activation of β-catenin in human testis-derived cells following oestrogen treatment has not previously been reported and is a major finding in understanding how oestrogen can influence gonad somatic cell fate. We have established that oestrogen can limit the bioavailability of SOX9 rapidly and lead to suppression of its downstream targets while also promoting the expression of β-catenin target genes such as *WNT4* and *FOXL2*. Here, we show that oestrogen can activate β-catenin within 30 min, and this occurs alongside the cytoplasmic retention of SOX9. It is well established that there exists antagonism between SOX9/pro-testis factors and β-catenin/pro-ovary factors in the gonad and we have demonstrated that oestrogen promotes the shift from SOX9 to β-catenin. We have further linked this into the role of the MAP3K1 cascade in mediating this shift in SOX9 and β-catenin through its activation, suggesting this pathway is a mechanism through which oestrogen influences gonad somatic cell fate. From these findings, we have defined a previously unknown mechanism through which oestrogen can promote a switch in gonad somatic cell fate and provided novel insights into the impacts of oestrogen exposure on the testis. We hypothesise that this is the ancestral mechanism that allows oestrogen to drive ovarian differentiation across vertebrates and is important for maintaining granulosa cell fate in adult mammals.

## 4. Materials and Methods

### 4.1. Cell Culture, Oestrogen Treatments, and SILAC

The human testis-derived embryonal carcinoma NTERA-2 clone D1 [NT2/D1] (cells were purchased from ATCC.org; ATCC CRL-1973) cell line shares many properties with Sertoli cells. They express *SOX9*, show activation of SOX9 target genes, suppression of female markers, and endogenously express oestrogen receptors, making them most akin to an adult Sertoli cell [57,58]. For stable isotope labelling with amino acids in cell culture (SILAC) [59], cells were maintained at 37 °C, 5% CO_2_ in Dulbeccos Modified Eagles Medium for SILAC (Thermo Fisher Scientific, Sydney, Australia) with 10% fetal bovine serum (Gibco, Sydney, Australia) and antibiotic-antimycotic (Gibco, Sydney, Australia). These cells were labeled with L-Lysine-2HCl, 13C6, 15N2 and L-Arginine-HCl, 13C6, 15N4 (Thermo Fisher Scientific, Waltham, MA, USA). Control cells were maintained as previously described [24,25]. Labelled cells were treated for 30 min (*n* = 4) or 48 h (*n* = 3) with 100 nM 17 α-ethynylestradiol (EE2; Sigma Aldrich, Sydney, Australia) dissolved in ethanol. EE2 was chosen due to its longer half-life in culture, its biological relevance given its widespread use and pervasiveness in our environment [60,61], and established use in our previous studies. The final concentration of ethanol added to culture was <0.05%. An identical amount of ethanol was added to control cells, which were grown in the absence of oestrogen.

### 4.2. Protein Digestion and Phosphopeptide Enrichment

Proteins were extracted from control and labelled NT2/D1 cells treated with EE2 for either 30 min (*n* = 4) or 48 h (*n* = 3). Each sample was lysed in RIPA buffer (25 mM Tris-HCl pH7.4, 150 mM NaCl, 0.1% (*w/v*) SDS, 1% (*v/v*) Triton X-100) supplemented with Halt Protease and Phosphatase Inhibitor Cocktail (Thermo Fisher Scientific, Sydney, Australia). Lysates were cleared by centrifugation at 13,000 rpm for 20 min at 4 °C. Proteins were precipitated with acetone, and subsequently re-dissolved in digestion buffer (8 M urea in 50 mM TEAB [pH 8.0]). Protein concentrations were measured using a Pierce Bradford Assay Kit (Thermo Fisher Scientific, Sydney, Australia). Protein samples (1 mg) from corresponding control and EE2 treated cells were mixed 1:1 and reduced, alkylated and digested (Pierce Trypsin Protease, Thermo Scientific, Sydney, Australia). An aliquot of 100 μg of the digested peptides were fractionated into 8 fractions using Pierce High pH Reversed-Phase Peptide Fractionation Kit (Thermo Fisher Scientific, Sydney, Australia) and used to determine the global proteome. The remaining peptides were acidified and purified by solid phased extraction using Oasis HLB cartridges (Waters, Sydney, Australia) and used for phosphopeptide enrichment using a titanium dioxide (TiO_2_) enrichment method [62]. Eluted phosphorylated peptides were freeze-dried before LC-MS/MS analysis.

### 4.3. LC-MS/MS

Samples were analysed by LC-MS/MS using the Q-Exactive Plus mass spectrometer (Thermo Scientific) fitted with nanoflow reversed-phase-HPLC (Ultimate 3000 RSLC, Dionex, CA, USA) as previously described [63].

### 4.4. Data Processing

All raw data were processed using MaxQuant [64] for SILAC-based approaches [65]. Database searches were conducted against the Swissprot reviewed *Homo Sapiens* database (www.uniprot.org, accessed on 5 April 2019). Default parameters in MaxQuant were used wherever applicable. False discovery rate (FDR) was set to 1% on both protein and peptide level and only peptides with a length of a minimum of 7 amino acids were considered. Carbamidomethylation of cysteine was set as fixed modification and oxidation of methionine and *N*-terminal acetylation were set as variable modifications. For phosphoproteomics data, phospho(STY) was included as variable modifications. The appropriate SILAC labels were selected and match between runs feature activated. Protein identification required a minimum of two peptides with at least one razor or unique peptide. Protein groups identified in MaxQuant were imported into Perseus [66] for further analysis. Results were filtered to remove potential contaminants, reverse hits, and hits only identified by site. For phosphoproteomic data, the phospho(STY)sites.txt file was imported into Perseus software for analysis. We removed any phosphopeptides with a localisation probability of <0.75. Normalised heavy/light (H/L) ratios were log_2_ transformed and represent the fold change of proteins following EE2 treatment. Values have been presented as averages for each treatment. Significance was determined by one sample T-test with Benjamin-Hochberg FDR adjusted *p* < 0.05. To analyse enrichment of Gene Ontology (GO) terms, proteins that significantly changed in abundance (*p* < 0.05, log_2_FC > or <0.25) were searched using the PANTHER database (http://www.pantherdb.org, accessed on 5 April 2020) [45] for biological processes and cellular components that were statistically enriched (Fisher’s exact test, FDR *p* < 0.05). Proteomic data obtained by mass spectrometry have been deposited in the ProteomeXchange Consortium via the PRIDE partner repository and are linked to the dataset identifier PXD026927.

### 4.5. Kinase-Substrate Analysis of the MAP3K1 and MAP3K4 Cascades

We performed kinase-substrate analysis to elucidate the overall activity of kinases involved in the MAP3K1 and MAP3K4 cascades. Substrate-kinase relationships were searched using a combination of Phosphomatics (https://www.phosphomatics.com, accessed on 5 April 2020) [67], Signor 2.0 (https://signor.uniroma2.it, accessed on 5 April 2020) [68], and PhosphoSitePlus (https://www.phosphosite.org, accessed on 5 April 2020) [69] to determine the upstream kinases responsible for significantly changed phosphosites. A comparison of the number of site- or protein-level changes for each kinase was also performed.

## Data Availability

The mass spectrometry proteomics data have been deposited to the ProteomeXchange Consortium via the PRIDE partner repository with the dataset identifier PXD026927.

## Supplementary Materials

Supplementary materials can be found at https://www.mdpi.com/article/10.3390/ijms221810046/s1.

## Abbreviations

ER	Oestrogen receptor
NT2/D1	NTERA-2 clone D1
SOX9	Sex-determining region Y box transcription factor 9
SRY	Sex-determining region Y
AMH	Anti-Mullerian hormone
FGF9	Fibroblast growth factor
PTGDS	Prostaglandin D synthase
FOXL2	Forkhead box L2
RSPO1	R-spondin 1
WNT4	Wnt family member 4
FST	Follistatin
ERK1/2	Extracellular regulated kinases 1/2
MAP3K	Mitogen-activated protein kinase kinase kinase
RHOA	Ras homolog family member A
ROCK	Rho-associated coiled coil containing protein kinase
GSK3β	Glycogen synthase kinase 3β
FRAT1	FRAT regulator of Wnt signalling pathway 1
GADD45γ	Growth arrest and DNA damage-inducible protein γ
PKA	Protein kinase A
AKT	AKT serine/threonine kinase
PAK1	p21 (RAC1) activated kinase 1
