# Supplementary material for: Oestrogen Activates the MAP3K1 Cascade and β-Catenin to Promote Granulosa-like Cell Fate in a Human Testis-Derived Cell Line
Source: Int J Mol Sci. 2021 Sep 17;22(18):10046. doi: 10.3390/ijms221810046 (PMC8471392; doi:10.3390/ijms221810046)
Supplement: Supplementary file 1 [file ijms-22-10046-s001.zip › ijms-1204025-supplementary.pdf]

Supplementary tables

Table S1. Fold enrichment and p-values of terms enriched in proteome following EE2 treatment reported in Figures 4 and 5.

PANTHER GO term	Fold enrichment	FDR-adjusted p-value	Timepoint
ribose phosphate biosynthetic process (GO:0046390)	24.65	4.91E-02	30 min
cytoskeleton organization (GO:0007010)	3.64	3.49E-03	30 min
Arp2/3 complex-mediated actin nucleation (GO:0034314)	17.12	1.30E-02	30 min
nucleotide-sugar metabolic process (GO:0009225)	12.84	2.20E-02	30 min
purine-containing compound metabolic process (GO:0072521)	10.27	3.68E-02	30 min
nucleoside triphosphate metabolic process (GO:0009141)	8.99	1.47E-02	30 min
actin filament polymerization (GO:0030041)	6.27	1.91E-02	30 min
monosaccharide metabolic process (GO:0005996)	4.11	4.39E-02	30 min
cellular component morphogenesis (GO:0032989)	3.33	4.82E-02	30 min
nuclear pore central transport channel (GO:0044613)	11.74	1.44E-02	30 min
protein-containing complex (GO:0032991)	1.56	1.46E-02	30 min
cytosolic ribosome (GO:0022626)	4.07	7.10E-03	30 min
ribonucleoprotein complex (GO:1990904)	3.17	1.40E-03	30 min
nucleolar part (GO:0044452)	5.48	5.70E-03	30 min
nucleolus (GO:0005730)	5.14	7.13E-06	30 min
cytoskeletal part (GO:0044430)	6.6	1.10E-03	30 min
actin cytoskeleton (GO:0015629)	4.57	7.28E-07	30 min
cytoskeleton (GO:0005856)	2.57	8.56E-05	30 min
spliceosomal snRNP complex (GO:0097525)	3.83	4.56E-02	30 min
nucleotide-sugar metabolic process	8.57	1.61E-02	48 hr

(GO:0009225)			
metabolic process (GO:0008152)	1.45	3.65E-07	48 hr
mRNA cis splicing, via spliceosome (GO:0045292)	7.95	3.97E-03	48 hr
RNA splicing (GO:0008380)	3.76	4.46E-06	48 hr
gene expression (GO:0010467)	1.46	5.60E-03	48 hr
ribosomal large subunit biogenesis (GO:0042273)	5.54	1.66E-02	48 hr
ribonucleoprotein complex biogenesis (GO:0022613)	2.98	8.48E-03	48 hr
cellular component organization or biogenesis (GO:0071840)	2.61	4.47E-03	48 hr
regulation of mRNA processing (GO:0050684)	4.42	5.81E-03	48 hr
chromatin organization (GO:0006325)	2.9	1.12E-02	48 hr
cellular protein localization (GO:0034613)	1.77	3.29E-03	48 hr
intracellular protein transport (GO:0006886)	1.73	1.64E-02	48 hr
transcriptional repressor complex (GO:0017053)	9.52	7.77E-03	48 hr
nuclear lumen (GO:0031981)	2.24	3.25E-05	48 hr
intracellular membrane-bounded organelle (GO:0043231)	1.53	1.14E-05	48 hr
protein-containing complex (GO:0032991)	1.45	5.56E-03	48 hr
proteasome core complex (GO:0005839)	5.44	4.12E-02	48 hr
nuclear speck (GO:0016607)	7.91	2.53E-04	48 hr
ribonucleoprotein complex (GO:1990904)	3.04	1.10E-05	48 hr
U2-type spliceosomal complex (GO:0005684)	4.73	3.43E-02	48 hr
preribosome (GO:0030684)	3.76	2.21E-03	48 hr
cell-cell junction (GO:0005911)	3.92	2.74E-03	48 hr
nucleolus (GO:0005730)	2.54	1.52E-02	48 hr
actin cytoskeleton (GO:0015629)	2.23	1.46E-02	48 hr
cytoskeleton (GO:0005856)	1.63	4.18E-02	48 hr
endoplasmic reticulum (GO:0005783)	2.17	1.09E-02	48 hr
vacuole (GO:0005773)	1.74	7.58E-05	48 hr
cytosol (GO:0005829)	1.72	5.76E-03	48 hr
plasma membrane (GO:0005886)	1.34	1.63E-02	48 hr

Table S2. Fold enrichment and p-values of terms enriched in phosphoproteome following EE2 treatment reported in Figures 4 and 5.

PANTHER GO term	Fold enrichment	FDR-adjusted p-value	Timepoint
regulation of supramolecular fiber organization (GO:1902903)	19.52	9.83E-03	30 min
regulation of cellular component organization (GO:0051128)	4.46	2.12E-05	30 min
regulation of RNA biosynthetic process (GO:2001141)	2.02	1.02E-02	30 min
regulation of metabolic process (GO:0019222)	1.99	9.59E-05	30 min
regulation of microtubule cytoskeleton organization (GO:0070507)	9.41	6.99E-03	30 min
regulation of cytoskeleton organization (GO:0051493)	6.39	5.88E-03	30 min
regulation of organelle organization (GO:0033043)	5.17	9.65E-04	30 min
microtubule depolymerization (GO:0007019)	11.56	1.03E-02	30 min
protein-containing complex disassembly (GO:0032984)	7.12	1.69E-02	30 min
gene expression (GO:0010467)	2.05	3.42E-07	30 min
transcription by RNA polymerase II (GO:0006366)	2.04	9.09E-04	30 min
regulation of mRNA processing (GO:0050684)	6.38	2.33E-03	30 min
RNA splicing (GO:0008380)	4.99	7.69E-06	30 min
RNA processing (GO:0006396)	4.71	4.94E-07	30 min
RNA catabolic process (GO:0006401)	4.04	4.08E-02	30 min
neuron development (GO:0048666)	3.83	1.83E-03	30 min
actin cytoskeleton organization (GO:0030036)	2.94	4.02E-02	30 min
mitotic nuclear division (GO:0140014)	2.94	2.15E-02	30 min
intracellular organelle (GO:0043229)	1.85	5.48E-07	30 min
ribonucleoprotein complex (GO:1990904)	3.2	1.57E-03	30 min
small nuclear ribonucleoprotein complex (GO:0030532)	4.05	4.89E-02	30 min

cytoskeleton (GO:0005856)	2.37	8.97E-04	30 min
microtubule cytoskeleton (GO:0015630)	2.62	3.99E-03	30 min
cell cycle (GO:0007049)	2.81	1.97E-06	48 hr
organelle organization (GO:0006996)	2.26	1.84E-04	48 hr
reproductive process (GO:0022414)	4.16	3.08E-02	48 hr
neuron development (GO:0048666)	3.06	1.85E-02	48 hr
regulation of cellular component organization (GO:0051128)	3.7	4.51E-04	48 hr
regulation of supramolecular fiber organization (GO:1902903)	18.01	8.54E-03	48 hr
regulation of RNA biosynthetic process (GO:2001141)	1.92	1.62E-02	48 hr
regulation of metabolic process (GO:0019222)	2.17	5.99E-07	48 hr
regulation of protein modification process (GO:0031399)	15.2	4.94E-02	48 hr
regulation of cytoskeleton organization (GO:0051493)	5.16	2.15E-02	48 hr
regulation of microtubule cytoskeleton organization (GO:0070507)	8.69	6.44E-03	48 hr
regulation of organelle organization (GO:0033043)	4.77	1.38E-03	48 hr
microtubule depolymerization (GO:0007019)	10.67	9.43E-03	48 hr
protein-containing complex disassembly (GO:0032984)	7.67	3.98E-03	48 hr
nuclear transport (GO:0051169)	7.85	9.31E-03	48 hr
regulation of mRNA processing (GO:0050684)	6.54	7.22E-04	48 hr
gene expression (GO:0010467)	2.09	1.50E-08	48 hr
RNA processing (GO:0006396)	4.18	4.41E-06	48 hr
RNA splicing (GO:0008380)	4.61	1.24E-05	48 hr
transcription by RNA polymerase II (GO:0006366)	1.84	5.50E-03	48 hr
negative regulation of cellular component organization (GO:0051129)	5.93	2.51E-02	48 hr
RNA catabolic process (GO:0006401)	4.66	5.62E-03	48 hr
peptidyl-lysine modification (GO:0018205)	4.05	3.42E-02	48 hr
chromosome organization (GO:0051276)	3.87	1.81E-04	48 hr

DNA metabolic process (GO:0006259)	3.41	3.16E-02	48 hr
mitotic cell cycle process (GO:1903047)	3.24	2.49E-03	48 hr
mitotic nuclear division (GO:0140014)	3.1	5.50E-03	48 hr
actin cytoskeleton organization (GO:0030036)	2.94	2.33E-02	48 hr
nuclear speck (GO:0016607)	7.79	1.12E-02	48 hr
transcription factor complex (GO:0005667)	5.5	3.59E-03	48 hr
actin filament (GO:0005884)	4.27	4.40E-02	48 hr
cell-cell junction (GO:0005911)	4.05	2.99E-02	48 hr
nuclear envelope (GO:0005635)	3.64	2.88E-02	48 hr
nucleolus (GO:0005730)	3.38	6.78E-03	48 hr
ribonucleoprotein complex (GO:1990904)	3.31	2.95E-04	48 hr
nuclear part (GO:0044428)	3	5.00E-11	48 hr
microtubule cytoskeleton (GO:0015630)	2.54	3.17E-03	48 hr
cytoskeleton (GO:0005856)	2.4	1.63E-04	48 hr
nuclear chromatin (GO:0000790)	2.12	1.72E-02	48 hr
chromosome (GO:0005694)	2.1	7.90E-03	48 hr
intracellular organelle (GO:0043229)	2.02	4.84E-11	48 hr
protein-containing complex (GO:0032991)	1.59	7.67E-03	48 hr

Table S3. Log$_2$ fold changes and p-values of proteins and phosphoproteins reported in Figure 6

Protein name (phosphosite)	Uniprot ID	30 min EE2 log$_2$FC	30 min EE2 p-value	48 hr EE2 log$_2$FC	48 hr EE2 p-value
CTNNB1	P35222			-0.311	0.021
CTNNB1 (S552)	P35222	0.551	0.033		
GSK3B	P49841	-0.220	0.085	-0.392	0.0153
KCTD1	Q719H9			-1.547	0.012
MAP2K1	Q02750			0.354	0.081
MAP2K2	P36507			0.153	0.025
MAP2K3	P46734			0.224	0.007
MAP2K6	P52564	-0.404	0.078		
MAP3K1 (S923)	Q13233	0.164	0.129	0.266	0.112
MAPK1 (Y187)	P28482	1.354	0.106		
MAPK14	Q16539			-0.216	0.032
PAK1	Q13153			0.455	0.007
PRKAR1A	P10644			-0.349	0.004
PRKAR1A (S83)	P10644	-0.673	0.003	-1.716	0.005
PRKAR1B	P31321			-1.047	0.011
PRKAR2A	P13861	-0.322	0.003	0.497	0.010
PRKAR2A (S99)	P13861	0.458	0.026	0.541	0.021
RHOA	P61586			0.345	0.002
ROCK1	Q13464			0.242	0.218
ROCK2	O75116	0.270	0.028	0.171	0.132

Table S4. Log_2 fold changes and p-values of phosphosites from substrate-kinase analyses after 30 minutes presented in Figure 6

Kinase	Kinase Uniprot ID	Substrate phosphosite	Substrate Uniprot ID	30 min EE2 log_2FC	30 min EE2 p-value
AKT1	P31749	CARHSP1 S52	Q9Y2V2	0.403	0.0153
AKT1	P31749	CTNNB1 S552	P35222	0.551	0.0331
AKT1	P31749	HTT S419	P42858	-0.253	0.0175
AKT1	P31749	PEA15 S116	Q15121	0.9	0.0031
GSK3B	P49841	RCAN1 S163	Q9UKA8	-2.6	0.0002
GSK3B	P49841	DPYSL3 S522	Q14195	-0.96	0.0082
GSK3B	P49841	EIF4EBP1 S37	Q13541	-0.64	0.0078
MAPK1	P28482	EIF4EBP1 S37	Q13541	-0.64	0.0078
MAPK1	P28482	PML S527	P29590	-0.96	0.0101
MAPK1	P28482	STMN1 S38	P16949	-0.53	0.0200
MAPK14	Q16539	EIF4EBP1 S37	Q13541	-0.64	0.0078
MAPK3	P27361	STMN1 S38	P16949	-0.53	0.0200
MAPK8	P45983	EIF4ENIF1 S587	Q9NRA8	-1.909	0.0334
MAPK8	P45983	STMN1 S38	P16949	-0.53	0.0200
PAK1	Q13153	CTNNB1 S552	P35222	0.551	0.0331
PAK1	Q13153	FLNA S2152	P21333	0.381	0.0069
PAK1	Q13153	STMN1 S16	P16949	0.7	0.0151
PRKACA	P17612	STMN1 S16	P16949	0.7	0.0151
PRKACA	P17612	SUFU S346	Q9UMX1	0.6	0.0204
PRKACA	P17612	NEDD4L S448	Q96PU5	-1.12	0.0159
PRKACA	P17612	FLNA S2152	P21333	0.381	0.0069

Table S5. Log2 fold changes and p-values of phosphosites from substrate-kinase analyses after 48 hours presented in Figure 6

Kinase	Kinase Uniprot ID	Substrate phosphosite	Substrate Uniprot ID	48 hr EE2 log_2FC	48 hr EE2 p-value
AKT1	P31749	PEA15 S116	Q15121	0.519	0.0418
AKT1	P31749	CARHSP1 S52	Q9Y2V2	0.478	0.0117
AKT1	P31749	CHEK1 S280	O14757	0.865	0.0041
GSK3B	P49841	RCAN1 S163	Q9UKA8	-0.335	0.0204
GSK3B	P49841	MYC T58	P01106	-1.122	0.0006
GSK3B	P49841	DPYSL3 S509	Q14195	0.23	0.0274
MAPK1	P28482	POLR2A S1920	P24928	-1.263	0.0229
MAPK1	P28482	POLR2A S1913	P24928	-1.302	0.0089
MAPK1	P28482	MYC S62	P01106	-1.109	0.0013
MAPK1	P28482	MYC T58	P01106	-1.122	0.0006
MAPK1	P28482	RPTOR S863	Q8N122	-0.16	0.0124
MAPK1	P28482	STMN1 S38	P16949	-1.16	0.0016
MAPK3	P27361	RPTOR S863	Q8N122	-0.16	0.0124
MAPK3	P27361	STMN1 S38	P16949	-1.16	0.0016
MAPK3	P27361	POLR2A S1920	P24928	-1.263	0.0229
MAPK3	P27361	POLR2A S1913	P24928	-1.302	0.0089
MAPK8	P45983	EIF4ENIF1 S587	Q9NRA8	-1.909	0.0302
MAPK8	P45983	HNRNPK S216	P61978	0.65	0.0084
MAPK8	P45983	MYC S62	P01106	-1.109	0.0013
MAPK8	P45983	STMN1 S38	P16949	-1.16	0.0016
PAK1	Q13153	STMN1 S38	P16949	-1.16	0.0016
PRKACA	P17612	CAD S1859	P27708	-0.925	0.0404
PRKACA	P17612	MAP2 S1782	P11137	-1.289	0.0053
